# Supplementary material for: Iterative improvement in the automatic modular design of robot swarms
Source: PeerJ Comput Sci. 2020 Dec 7;6:e322. doi: 10.7717/peerj-cs.322 (PMC7924708; doi:10.7717/peerj-cs.322)
Supplement: Supplemental Information 3 [file peerj-cs-06-322-s003.zip › argos3/doc/api/standalone/a00407.html]

ARGoS: core/utility/string\_utilities.h File Reference


- Main Page
- Related Pages
- Namespaces
- Classes
- Files

- File List
- File Members

# core/utility/string\_utilities.h File Reference

`#include <argos3/core/utility/configuration/argos_exception.h>`  
`#include <argos3/core/utility/datatypes/datatypes.h>`  
`#include <string>`  
`#include <vector>`  
`#include <sstream>`  

Include dependency graph for string\_utilities.h:

This graph shows which files directly or indirectly include this file:

Go to the source code of this file.

|  |  |
| --- | --- |
| Namespaces | |
| namespace | argos |

|  |  |
| --- | --- |
|  | The namespace containing all the ARGoS related code. |

| Functions | |
| template<typename T > | |
| std::string | argos::ToString (const T &t\_value) |
|  | Converts the given parameter to a `std::string`. |
| template<typename T > | |
| T | argos::FromString (const std::string &str\_value) |
|  | Converts the given `std::string` parameter to the wanted type. |
| template<typename T > | |
| void | argos::ParseValues (std::istream &str\_input, UInt32 un\_num\_fields, T \*pt\_field\_buffer, const char ch\_delimiter= '\n') |
| template<typename T > | |
| void | argos::ParseValues (const std::string &str\_input, const UInt32 un\_num\_fields, T \*pt\_field\_buffer, const char ch\_delimiter= '\n') |
| void | argos::Tokenize (const std::string &str\_string, std::vector< std::string > &vec\_tokens, const std::string &str\_delimiters=" ") |
|  | Tokenizes the given string according to the wanted delimiters (by default just a " "). |
| std::string | argos::StringToUpperCase (const std::string &str\_string) |
|  | Converts a string to upper case. |
| std::string | argos::StringToLowerCase (const std::string &str\_string) |
|  | Converts a string to lower case. |
| void | argos::Replace (std::string &str\_buffer, const std::string &str\_original, const std::string &str\_new) |
|  | Searches into str\_buffer for occurrences of str\_original and substitutes them with str\_new. |
| bool | argos::MatchPattern (const std::string &str\_input, const std::string &str\_pattern) |
|  | Returns `true` if str\_pattern is matched by str\_input. |
| std::string & | argos::ExpandEnvVariables (std::string &str\_buffer) |
|  | Searches into str\_buffer for occurrences of an environment variable of the form $VAR and substitutes them with the value of the variable. |

---

Generated on 10 Jul 2018 for ARGoS by 
 1.6.1
